# Co‐Producing Resources to Help Improve Access to Primary Care for Young People With Attention Deficit Hyperactivity Disorder

**DOI:** 10.1111/hex.70200

**Published:** 2025-04-29

**Authors:** Rebecca Gudka, Anita Salimi, Rachel Gaywood, Dale Hendrick, Kieran Becker, Oliver Medzinskii, Faraz Mughal, G. J. Melendez‐Torres, Jane Smith, Tamsin Newlove‐Delgado, Anna Price

**Affiliations:** ^1^ University of Exeter Medical School Exeter UK; ^2^ NHS Devon Integrated Care Board Exeter UK; ^3^ Devon Adult Autism and ADHD Service Exeter UK; ^4^ Department of Computer Sciences University of Exeter Exeter UK; ^5^ School of Medicine, Keele University Staffordshire UK

**Keywords:** attention deficit hyperactivity disorder, co‐production, health policy, health services, primary care

## Abstract

**Background:**

Attention deficit hyperactivity disorder (ADHD) is a common neurodevelopmental disorder resulting in negative long‐term outcomes if untreated. Pathways to healthcare in the United Kingdom are complex, especially for those aged 16–25 transitioning between child and adult mental health services. We aimed to co‐produce evidence‐informed resources to improve accessibility of primary care for young people with ADHD.

**Methods:**

We utilised co‐production principles from the National Institute for Health and Care Research and findings from recent research to create evidence‐informed resources which collate experiences of multiple stakeholders. Lived experience advisors (young people aged 16–25 with ADHD and their supporters) and healthcare professionals were recruited through previous research engagement and local collaborations. Research partners engaged in workshops or individual meetings to agree priorities, content, and language/visual appearance for outputs.

**Results:**

Lived experience advisors (7), healthcare professionals (5), and researchers (3) co‐produced a downloadable resource for young people and their supporters. The resource identifies key stages on ADHD healthcare pathways, common barriers, and top tips recommended by people with lived experience, and verified by healthcare professionals. Key messages for primary care professionals were co‐produced.

**Conclusion:**

Co‐produced resources can help address barriers to accessing ADHD treatment and support via stretched NHS services. Collaborative working also highlighted the need for national policy change to alleviate pressures faced by healthcare professionals and patients.

**Patient and Public Contribution:**

Two research advisory groups (RAGs) of healthcare professionals and lived experience advisors informed research methods and presentation of results. RAG members participated in co‐production workshops, contributed to authorship, and disseminated outputs.

## 1 Background

### 1.1 Attention Deficit Hyperactivity Disorder (ADHD)

ADHD is the most common neurodevelopmental disorder, affecting approximately 5%–7% children and adolescents, and 2%–5% adults globally [1, 2, 3, 4]. ADHD is characterised by hyperactivity, impulsivity and/or inattention which leads to impairment in daily‐life functioning. Individuals with ADHD often experience negative long‐term outcomes in multiple areas including education, employment, and financial management, and are at increased risk of mental health comorbidities [5, 6]. An estimated 40% of individuals diagnosed with ADHD in childhood or adolescence continue to experience symptoms into adulthood [7]. Both short‐ and long‐term outcomes experienced by individuals with ADHD can be improved with treatment, which commonly consists of medication but can include non‐pharmacological support such as psychosocial interventions [8, 9, 10]. However, an effective approach to long‐term ADHD management also requires regular reviews to ensure that individuals receive adequate access to support throughout the life‐span, and through changing environments and health‐needs [11].

Healthcare pathways for ADHD in the United Kingdom are complex and involve many steps. General practitioners (GPs) refer patients with suspected ADHD to specialist services for diagnostic assessment and are subsequently responsible for routine prescribing and monitoring of ADHD medication. GPs need support from specialist services to carry this out. Shared‐care agreements are protocols which support safe and effective collaboration between specialist and primary care settings. But results from a systematic review and cross‐sectional survey indicate that shared care components do not work consistently, leaving young people unable to access required medication [12, 13]. Specialist services are responsible for assessment and diagnosis, medication titration, and delivering non‐pharmacological interventions including psychoeducation, where available [14]. For individuals with ADHD, accessing healthcare and support for associated mental health conditions, such as depression or anxiety, often requires navigating separate pathways and services. Qualitative research has identified this process as an “uphill struggle” for individuals with ADHD [15]. Our recent qualitative work highlights that accessing or delivering ADHD care is laborious for individuals with ADHD and healthcare professionals, respectively [16].

Structural divisions between child and adult health services create additional barriers for young people with ADHD aged 16–25 years old [17]. These challenges arise when children transitioning into adulthood are vulnerable to mental health crises and to developing long‐term mental health conditions [18]. UK guidelines recommend putting systems in place to support the transition between services for young patients with ADHD, including fully informing young people about adult services, and conducting assessments of their personal, educational and social functioning post‐transition [19]. Positive condition management during this period can have a reduce the risks of negative outcomes, and help individuals with ADHD to thrive [19]. However, a national surveillance study shows that fewer than one in four people who required medication for their ADHD successfully made the transition between services, resulting in extended gaps in medication prescriptions [20]. In addition to national challenges in accessing and transitioning to adult services, young people are likely to switch general practices due to moving to new areas, which could disrupt access to ADHD medication due to regional variation in prescribing via primary care [16, 21].

### 1.2 MAP Study

Awareness of the challenges faced by young people with ADHD is growing, as are the pressures faced by GPs to provide adequate ADHD healthcare, with primary care being the first port of call [14]. However, little research explores experiences in primary care from multiple stakeholder perspectives, and there are limited practical recommendations for supporting healthcare transitions. The NIHR‐funded Managing young people with ADHD in Primary care (MAP) study incorporates quantitative and qualitative data from a broad range of stakeholders to address the evidence gap on current primary care provision for young people with ADHD [16, 21, 22].

The project aimed to explore experiences of accessing and/or delivering primary care for ADHD in England. Two research advisory groups (RAGs) were consulted throughout on methods, presentation of findings, and development of future research questions. The Young person RAG included young people with ADHD aged between 16 and 25 and supporters of young people with ADHD; the practitioner RAG consisted of healthcare professionals involved in the delivery of ADHD care, including GPs, commissioners and other roles such as an occupational therapist and non‐medical nurse prescriber.

As part of the MAP study, we conducted a national survey of over 700 participants from across England [21], and a qualitative study to gain a deeper understanding of these results [16]. Key findings highlighted limited access to support for ADHD, with 45% of a population sample of commissioners reporting waiting times of 2 years or more for specialist services [21]. Many patients reported seeking private diagnoses due to lack of provision, then facing difficulties obtaining NHS prescriptions via their GPs [16, 21]. Participants indicated that reasonable adjustments in general practice could improve access to care for young people with ADHD [16]. Reasonable adjustments (changes made to a service to remove or reduce disadvantages faced) under the Equalities Act (2010) could be a way of helping patients with ADHD to access care, without placing undue strain on stretched services [16, 23]. MAP findings are summarised as an infographic in Appendix S1.

Findings from the MAP survey and qualitative studies add to an evidence‐base (as outlined above), which highlights the negative consequences of inadequacies in primary care systems for ADHD, both for patients and professionals. In this paper we present the final work package of the MAP study, in which researchers, experts by experience, and healthcare professionals worked together to synthesise research evidence to co‐produce guidance and resources to improve access to primary care in the United Kingdom.

### 1.3 Co‐Production

Co‐production is defined by the National Institute for Health and Care Research (NIHR) as a way of working that involves people using health services, carers, practitioners, and the wider community as active partners rather than passive recipients of research [24, 25]. The Five Year Forward View for Mental Health [26] called for development of evidence‐based approaches to co‐production in health service commissioning. The NHS Long Term Plan [27] emphasises the importance of working collaboratively to find solutions to address unmet health and social care needs. Therefore, this research was designed to involve stakeholders who could contribute varied knowledge and experience throughout the research process, with the aim of increasing quality, focussing on relevant issues, and co‐producing outputs that are deliverable in practice [28]. Outputs are intended to be co‐produced and evidence‐informed, to maximise the potential for impact and change.

### 1.4 Aims

The aim of this study is to co‐produce evidence‐informed resources to improve the delivery and accessibility of primary care for young people aged 16–25 with ADHD. Desired outputs of this study are:

- Information resources for young people with ADHD and their supporters
- Key messages and resources for primary care professionals (e.g., GPs) about delivering ADHD support, based on MAP study findings.

## 2 Methods

### 2.1 Research Partners

Research partners were recruited through the MAP RAGs, via a regular e‐newsletter and RAG meetings. RAG members were approached first as they were already engaged with the research and had a good understanding of the study background. After the initial recruitment, additional invitations were sent to people previously involved in projects aligned with the Science of ADHD and Neurodiversity (SAND) collaboration at the University of Exeter and who had consented to future contact regarding research opportunities. Individuals interested in participating provided demographic information which informed purposive sampling. Two researchers reviewed this information, then invited a sample designed to be diverse as possible in terms of role, gender, ethnic group and NHS region. Research partners were: four young people (aged 16–25) with ADHD, three parents or guardians (henceforth referred to as supporters) of young people with ADHD, five healthcare professionals, and three ADHD research representatives from the University of Exeter. The term *people with lived experience* is used to describe young people with ADHD and their supporters as a group. Table 1 details participant characteristics.

**Table 1:** Participant characteristics.

Participant ID	Main reported role	Gender	Ethnic group	NHS region
LE01	Supporter of young person with ADHD	Female	White	South East
LE02	Young person with ADHD	Female	Asian	Midlands
LE03	Supporter of young person with ADHD	Female	White	South West
LE04	Young person with ADHD	Male	White	South West
LE05	Supporter of young person with ADHD	Male	White	South West
LE06	Young person with ADHD	Female	Black	North West
LE07^a^	Young person with ADHD	Male	White	South West
HP01	GP and Commissioner	Female	White	South West
HP02	Occupational therapist (Mental health & Wellbeing)	Female	White	South West
HP03	Non‐medical nurse prescriber (Adult Autism & ADHD Service)	Male	White	South West
HP04	Academic GP	Male	Asian	Midlands
HP05	GP	Female	White	Midlands
RE01	Research Assistant	Female	Mixed	South West
RE02	Research Intern	Male	White	South West
RE03	Research Intern	Male	White	South West

^a^ LE07 was recruited after initial workshops for Phase 3.

Once recruitment targets were reached, research partners agreed on suitable dates and times of workshops. Research partners were provided with information sheets and given the opportunity to ask questions regarding their participation before providing informed consent. At the end of the workshops, research partners were asked if they were happy to be contacted again for further participation and output creation. Following low response rates to this request, one more participant (LE07) was recruited for the final phase of participation via the SAND collaboration.

Research partners were compensated for their time with £25 vouchers and given the opportunity to take part in writing‐up the results of the workshops in academic publications and to share findings at conferences or seminars.

### 2.2 Key Principles of Co‐Production

The practice of co‐production, as defined by NIHR [24] is characterised by key principles of:

Sharing of power
Including all perspectives and skills
Respecting and valuing the knowledge of all those working together on the project
Reciprocity
Building and maintaining relationships

To uphold these principles, research partners began each workshop with an opportunity to reflect on how each member could contribute to the project. Thus, encouraging the principle of respecting the knowledge of all partners. Participants were regarded as equal partners, with researchers acknowledged for in‐depth knowledge of the evidence‐base, young people with ADHD and their supporters acknowledged for lived experience and expertise in accessing care in real‐world settings, and healthcare professionals acknowledged for their expertise in primary care delivery. This approach enabled sharing of power, countering power‐imbalances created by using terms such as “researcher” and “participant”. Removing labels and focussing on skills brought by individual partners also supported the principle of including all perspectives and skills. Reciprocity and building of relationships were operationalised via an informal tone in meetings, and inclusion of partners throughout the research cycle, including during writing‐up, publishing and dissemination of results and outputs.

### 2.3 Phase 1: Designing Co‐Production Activities

**Figure 1:**
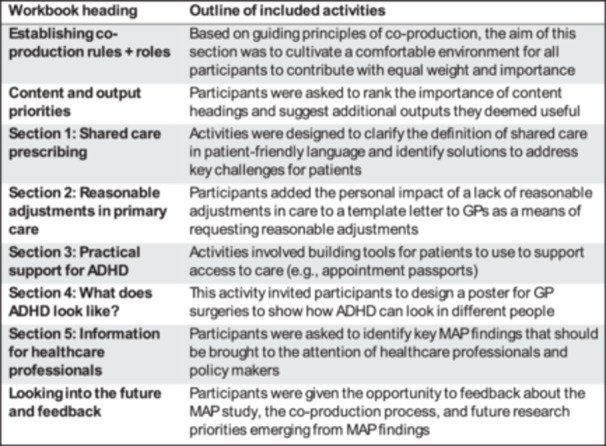
Workbook headings and a brief outline of the activities.

**Figure 2:**
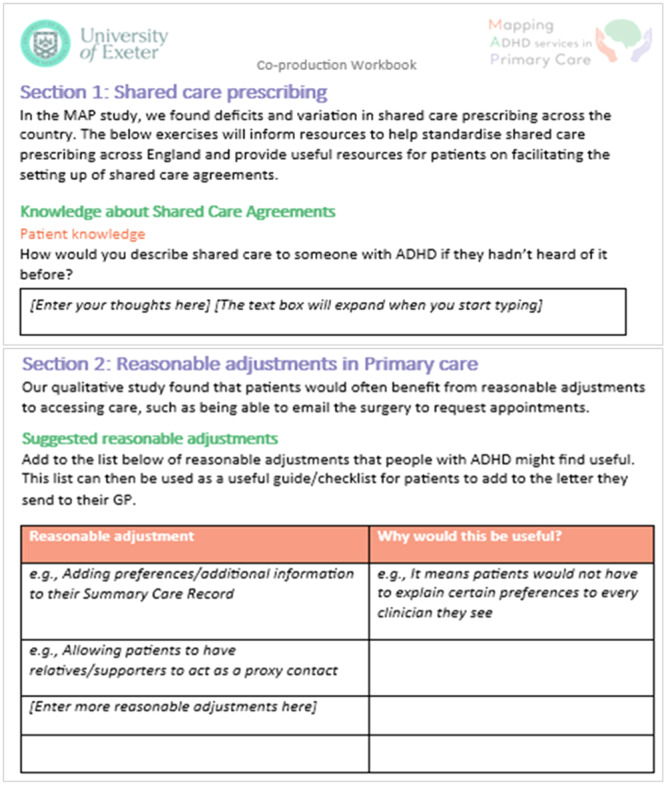
Screenshots of workbook activities.

RE01 summarised key findings from the MAP national survey and qualitative study to produce an infographic and workbook for research partners (Appendix S1). Co‐production activities centred around this workbook, with partners asked to read through it before workshops and activities completed then discussed during the workshop. The workbook content and structure were shared with MAP RAGs in RAG meetings, and feedback on activities was used to adapt the design of the workbook and workshop activities. These are detailed in Figure 1, with examples of workbook activities included in Figure 2.

### 2.4 Phase 2: Designing Co‐Production Outputs

As protocolised, this research was undertaken from within a critical realist stance, and founded on the assumption that perceptions of the nature of the world are shaped by experiences and observations [22, 29, 30]. This philosophical approach is compatible with co‐production methodology, and appropriate for applied health services research [30].

The workbook and infographic were sent to research partners, who were asked to read through the resources before meetings and attempt to fill in sections, to help them prepare for the online meetings. In email communications before the meetings, emphasis was placed on research partners only completing activities they felt able to. Reminders were provided that the group would work through each section together during the workshop as needed. A reminder email or text (depending on research partner communication preferences) was sent a day before the online workshop, along with a reminder to reach out with any questions about the workbook or how the workshop would be run.

Two online workshops were conducted, each lasting approximately 1.5 h. One workshop was held for people with lived experience of ADHD with five research partners, and another for healthcare professionals with two research partners. During the workshops, workbook activities were discussed in the same order that they appeared in the workbook. One person with lived experience and three healthcare professionals were unable to attend the workshops, so they either met separately with a researcher or filled in the workbook independently and returned it via email. During individual meetings partners discussed the workbook and reflected on workshop feedback.

The workshops were recorded and transcribed verbatim, and researchers kept detailed records of reflections on workshop content. Researchers discussed the workshops after each one to reflect on key findings and content. Content from the workshops, meetings and workbooks were transformed by RE01 into outputs. All workbook responses, researcher reflections, and relevant quotes from workshop transcripts were combined into a single ‘master’ workbook. The data for each section in the workbook was grouped based on similar experiences or sentiment.

Relevant and prioritised sections of the workbook, as determined by research partners in an activity at the start of the workbook, were moved to a PowerPoint file and reformatted to the first iteration of the downloadable resource for young people with ADHD and their supporters. Key messages for healthcare professionals were distilled from the master workbook and moved to a Word document, where they were grouped and reformatted. Where possible wording provided by research partners was retained, to reflect intended meaning, unless content was confusing or not concise. RE01 highlighted any adapted content and requested additional feedback from research partners on these changes during Phase 3. The first draft of the downloadable resource and messages for healthcare professionals were sent to research partners for further feedback in Phase 3.

### 2.5 Phase 3: Refining Co‐Production Outputs

Outputs were shared with those research partners who had indicated that they would like to feedback on the co‐production outputs, and with LE07, who was recruited after the workshops were concluded. These research partners fed‐back on content to ensure it reflected their views, and on the visual appearance of the outputs. Recommended changes were applied to the outputs by RE01.

Final outputs were shared with all research partners following the final stage of co‐production to ask them to share with their networks.

## 3 Results

Here we present the findings from our co‐production work, detailing reflections on the co‐production process and resulting outputs.

### 3.1 Co‐Production Process

Table 1 shows participant characteristics. We reached our target sample sizes, and our sample was representative with regards to role, gender, ethnicity, and NHS region. However, due to participant time constraints, healthcare professionals had to be consulted in separate meetings, so that the HP workshop only consisted of two research partners.

“Unfortunately, due to current workload demand I did not feel I had enough time to give more in depth responses and answers to some of the sessions or to the workbook.”

HP03

Feedback from research partners about taking part in the process was mostly positive. However, several noted that the long workbook was intimidating to work through alone. Positive workbook comments included that it created a helpful structure for the workshops and that working through it together enabled partners to bounce ideas off one another and combine their individual and varied experiences into one place. This helped to identify solutions with the potential to work for a broader audience.

“Well, I found today's discussion absolutely excellent. I've found the workbook and given that I haven't got ADHD, I found it quite big”

LE03

“It's really helpful to go through [the workbook] together and also other people's ideas are really helpful.”

LE02

The co‐production process was valuable for researchers to gain a better understanding of the potential impact of the MAP study findings on stakeholders. Research partners were able to provide their perspectives on challenges within primary care and offer their opinions on possible solutions and recommendations, informed by research evidence. Stakeholders reported feeling valued because their suggestions and opinions were held in the same regard as other research partners, regardless of role. The ability to share experiences with peers who may have had similar experiences also allowed research partners to feel validated. Some believed that sharing their negative experiences might benefit others in the future.

“I felt that my views and suggestions were validated and listened to and that the researchers really valued my input.”

HP03

Both lived experience and healthcare professional research partners stated that it was difficult and saddening to hear real life examples of ways the NHS is currently under‐resourced and how ADHD is still poorly understood by some clinicians. But reflected it was beneficial to hear insights from other perspectives, which they said they would be able to use when accessing/delivering care.

“I enjoyed being able to give the voice of GPs managing ADHD on daily basis. I also valued the opportunity to give a perspective from the ICB [Integrated Care Board]. At times I continue to feel frustrated that there is still so much work to be done for ADHD.”

HP01

When written responses to the workbook from each participant, researcher reflections, and quotes from workshop transcripts were combined into a master workbook, researchers were able to identify and group patterns of similar suggestions. Where possible, the wording used in each output directly reflects terms used by research partners (e.g., people with lived experience or healthcare professionals), with changes made only to improve clarity for the reader. Outputs were further refined during Phase 3, in which the resources were sent to research partners for suggestions and edits to wording and formatting.

**Figure 3:**
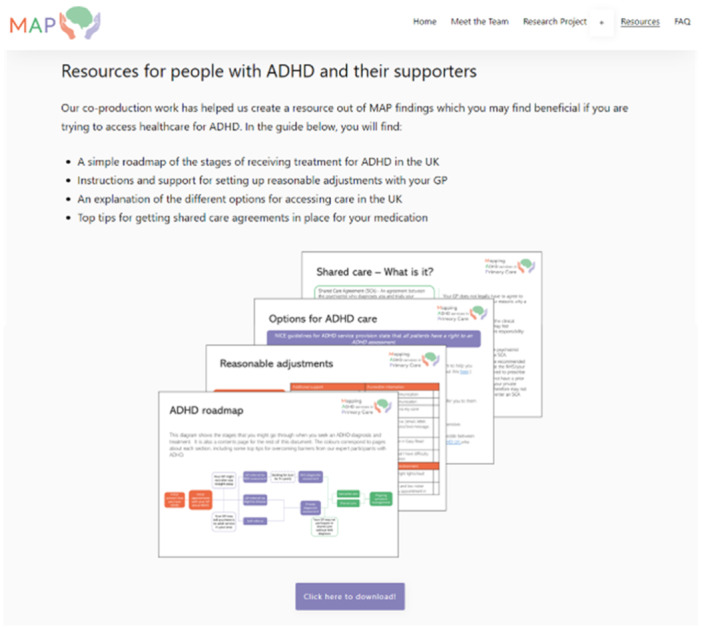
Screenshot of MAP website, where people can download co‐produced resource.

The finished outputs are hosted on the MAP study website (see Figure 3), and the SAND website for people to download. In addition to outputs for people with lived experience and healthcare professionals (described below), research partners delivered a presentation about their experiences of co‐production to the University of Exeter's Collaboration for Academic Primary Care (APEx). This presentation shared with researchers the importance of co‐producing outputs with stakeholders, common barriers to conducting this type of research, and lessons learned from MAP co‐production.

### 3.2 Outputs for People With Lived Experience

The completed workbooks and workshop contents which were relevant to young people with ADHD and their supporters have been developed into a downloadable resource. This can be viewed in Appendix S2.

The resource contains a “roadmap” of ADHD care pathways, which highlights points on the pathway which research partners and MAP findings indicated were places that many people come across challenges. These challenges form the headings for subsequent pages which present tools, resources, and tips for patients for overcoming barriers to care. The order and structure of the resource was determined by a workbook priority setting exercise and from sections that had the most engagement from research partners during workshops. The content is directly from people with lived experience and was verified by healthcare professionals to make sure that recommendations were actionable and appropriate to share with patients. The collaborative design process helped to ensure resources align with lived experience and healthcare professional expectations (as represented in this study). Researchers then added information from verified sources and MAP findings and structured the resource to maximise accessibility. Changes made during Phase 3 included adapting wording, adding icons and images, and changing the background colour and formatting.

### 3.3 Outputs for Healthcare Professionals

A key finding from this co‐production work is that healthcare professionals “*are feeling a bit broken*”—HP01. Healthcare professional research partners fed back that the initial language used to deliver key messages to healthcare professionals made them feel that their voices were not being heard and that they felt blamed for the negative consequences faced by young people. Time was taken during the workshop to ensure that language used throughout the rest of the process and in the below recommendations was more acceptable to healthcare professionals.

People with lived experience stressed that it was important for healthcare professionals to understand the impact of their interactions on patients’ lives. All research partners agreed that individual healthcare professionals should not be expected to overhaul their routines or practice. However, lived experience partners spoke about the negative consequences of stigma faced by patients and emphasised that positive communication should be at the forefront of key messages for healthcare professionals.

Below are five recommendations for healthcare professionals generated by research partners. These are: *strategies to support informed decision making by patients, using digital notes so that patients do not need to repeat their histories, increasing awareness of preconceptions and beliefs around ADHD, working towards equitable access for all patients with ADHD, and providing a structured space during appointments in which patients can address healthcare concerns*.

#### 3.3.1 Informed Decisions

We encourage healthcare professionals to inform patients about prescription policies and shared‐care agreements with private providers before referral for ADHD diagnostic assessments. It is important to understand the profound impact not receiving treatment can have on young people and their supporters’ lives. While healthcare professionals must adhere to practice policies and may be unable to prescribe to patients with private diagnoses, explaining these limitations to patients beforehand helps them to make informed decisions. Accessible information available on practice websites and from practice managers, clinical staff and administrators can help to avoid unintended consequences of patients being left without access to NHS prescriptions and monitoring.

“It can be incredibly expensive trying to fill private prescriptions if you meet a barrier with shared care.”

LE01

“I saved up for quite a while to get that diagnosis, and I know I would be heartbroken if I went to a GP, and they just rejected [my diagnosis]. It feels like you either have to pick between money or time, and time is something you'll never get back, but potentially you'll get money back.”

LE02

#### 3.3.2 “Tell Us Once”

Individuals with ADHD may find it challenging to repeat their histories to new healthcare professionals if they cannot see the same GP consistently. Making use of digital notes and clinical coding to implement a “tell us once” policy, so that patient histories are accurately and concisely described, can ease transition between healthcare professionals for patients.

#### 3.3.3 Preconceptions and Beliefs

By acknowledging and addressing preconceptions and stigma which may negatively impact individuals with ADHD, healthcare professionals may be able to create a more supportive and understanding environment. Research partners commonly brought up the following two points that they want healthcare professionals to understand. These relate to the different ways ADHD can present across the lifespan and with different populations, and to the fact patients learning about ADHD can most usefully be viewed as a positive attempt to understand and self‐manage their condition.

− Heterogeneous presentations of ADHD across populations, especially those which have non‐typical presentations (e.g., female patients diagnosed at a later age). Patients may have developed strategies to mask or cope with ADHD which means they may appear to be coping with symptoms.
  “Patients who look like they're coping may still have ADHD… they will have found ways of coping with it, but believe them when they say that they [believe that they have] ADHD”
  LE03
− The tendency to hyperfocus on learning about ADHD may help patients to manage anxiety or self‐manage their ADHD.

“It's important that GPs are aware that a [patient with ADHD] is already an expert in whatever it is that's wrong with them… It's not that they think that they are superior.”

LE03

#### 3.3.4 Equity, Not Equality

Characteristics of ADHD can hinder patient attendance and engagement with appointments. Offering accommodations like a 5‐min grace period for patients with ADHD can ensure greater and more consistent access to healthcare. Promoting fairness despite characteristics of ADHD, which may create barriers to access.

In addition to findings about patient‐healthcare professional interactions, both groups of research partners suggested that waiting rooms are a potential practical barrier to accessing care, which often feels overlooked. Consideration of creating quiet spaces for patients who were waiting might reduce barriers to primary care.

#### 3.3.5 Taking Up Space

Patient‐led appointments, where patients control the direction of conversations, and have time to explain themselves, can benefit individuals with ADHD. For example, allowing patients to explain their reasons for booking an appointment, rather than asking questions which may distract them, or taking the consultation in a direction patients do not feel is relevant. This may require longer appointments, which would count as a reasonable adjustment.

“[I find appointments] very doctor led and… it's very easy for a person with ADHD to get sidetracked. There's been a lot of times, for example, I've left an appointment and I felt like I've not been able to discuss the specific thing I wanted to bring up.”

LE02

## 4 Discussion

### 4.1 Summary

Using co‐production methods informed by a critical realist perspective this study aimed to develop resources to support young people with ADHD in accessing primary care and to produce key messages for healthcare professionals. Engaging a diverse range of stakeholders, including healthcare professionals and people with lived experience of ADHD, we collaboratively explored the types of support and guidance that might be produced, where they could be directed, and formats most beneficial to stakeholders. Our findings may enhance the ability of both service users and professionals to access appropriate support, improving access to primary care for young people with ADHD.

### 4.2 Developing Co‐Production Methodology

While key principles of co‐production research including sharing of power, building trusting relationships, and including diverse perspective have been clearly articulated [24, 31], the operationalisation of co‐production methodology in applied health research is less well clearly defined [31]. Research indicates that rather than following an ‘off‐the‐shelf’ approach, methods need to be context‐driven, appropriate for the research question and consider the specific needs (e.g. characteristics, life‐stage, challenges) of those involved [32]. Methods for delivering co‐production research working with individuals with ADHD, considering their related attentional and organisational needs, are not presented in a repeatable or implementable manner [33]. This research was shaped by the patient and public engagement work underpinning delivery of the broader MAP study and was delivered in line with MAP ethical approvals. It was delivered by a team with experience of collaborative working involving researchers, individuals with ADHD, and healthcare professionals. Methods used were informed by a critical realist stance and facilitated by researchers experienced in qualitative research methods, including reflective thematic analysis, which helped us to find creative ways of delivering this research and sharing findings [34, 35]. This publication aims to progress co‐production research involving individuals with ADHD by clearly communicating methods used and lessons learned.

### 4.3 Extending the Impact of “The Failure of Healthcare”

Previous research has indicated a “failure of healthcare” for young people with ADHD [36]. Findings from this co‐production point to a broader issue. That the healthcare system is not only failing to meet needs of individuals with ADHD, but that it is also placing healthcare professionals working in general practice under high levels of stress, as they struggle to deliver care and support for young people with ADHD in a system under pressure [13, 16]. While our understanding and knowledge of ADHD has improved over the past few decades, the healthcare system is out of date with advancements in our understanding of patient needs and under‐resourced for the current demand on service [14]. In this paper, we make recommendations for healthcare professionals based on an understanding of challenges young people with ADHD face, intended as a temporary solution to improve delivery of care for patients with ADHD. However, we stress that individuals and practices cannot meet increasing demand, deliver additional care, and undertake ADHD‐specific training to increase knowledge, without proportionate funding and systemic reforms, such as standardising ADHD healthcare nationally. Primary Care Networks and Integrated Care Boards do not have the resources or support needed to transform care for this underserved population without national policy change.

### 4.4 Digitising Outputs

MAP [16, 21], and CATCh‐uS study [37] findings, alongside wider research, indicate an opportunity for digital technologies to improve access to care [38, 39]. Previous research emphasises the importance of co‐producing digital health‐information resources with active involvement of all stakeholders in development, design, and subsequent evaluation [40]. In addition, it is crucial to understand how digital resources will integrate into current care pathways. A broad approach is needed to ensure digital healthcare information is curated, trusted, and easily accessed via trusted systems, such as the NHS England website, or GP practice IT systems. This foundational co‐production work is a first step towards creating digital resources for young people with ADHD and includes and exploration of content and delivery preferences of stakeholders.

### 4.5 Recommendations for Co‐Production With Stakeholder Groups

Previous research indicates that involving stakeholders in design processes can contribute to more useful and meaningful resources and interventions [28, 41]. This study also highlights the importance of co‐production with stakeholder groups in generating meaningful insights and recommendations.

Similarly to other research, we found time was a barrier to participation in co‐production activities [42]. One way we overcame this was by offering flexibility in the mode of delivery for workshops (i.e., allowing research partners to send completed workbooks if they could not attend, or having one‐to‐one meetings to discuss the workbook). This proved beneficial, allowing for broader participation, and accommodating differing schedules, which is particularly important when considering populations of both healthcare professionals and individuals with ADHD. Unfortunately, the workshop for healthcare professionals being poorly attended, leaving research partners feeling that the discussions during this meeting would have benefitted from a wider range of experiences and backgrounds.

Another recommendation to overcome time‐pressures faced by research partners is using virtual‐meeting environments [42]. Our virtual‐workshop environment offered convenience and accessibility for research partners, who could join from the comfort and privacy of their own homes. Online research can improve geographic diversity among research partners and offer cost‐effectiveness and flexibility, however there may also be disadvantages for those living in homes which are not comfortable or private, or which lack suitable devices and connectivity. Hence the offering virtual‐only options should be carefully considered in terms of impact on underserved groups [43, 44]. In this research, we successfully gained perspectives from stakeholders located in four of the seven NHS regions through virtual working.

The workbook was designed to facilitate workshop structure, which helped keep meetings to time and to ensure discussions remained on topic. However, feedback from research partners indicated that the workbooks contained an excessive amount of text, which may have contributed to a lack of engagement in Phase 3. In future, we recommend using tools to retain structure in meetings which are more concise, and making use of tools such as built‐in polls in Microsoft teams, as suggested by Ward et al [42].

Despite low retention rates for Phase 3, quality of engagement and contributions was high. During workshops and meetings, and in completed workbooks, research partners went into detail and depth to complete each activity. Accessing the workbook before workshops and being able to return this after the workshops gave research partners time to reflect on their own responses, share learning with others, and then reflect on responses from across the group. Benefits of this process were clear in the returned workbooks, for example when research partners mentioned other partners in their answers. These findings reflect those from previous research which focussed on the virtual delivery of co‐production methods [45, 46]. However, in contrast to Benson et al., we found that complex tasks where research partners had to draw images and insert their ideas into pre‐formatted letters were less enjoyable and led to less engagement [45]. This may highlight challenges of working with a population with ADHD, who can struggle to follow detailed instructions and maintain attention during complex tasks, although it could also reflect the digital format used.

### 4.6 Strengths and Limitations

This study benefitted from insights and perspectives from multiple stakeholders. We heard from people with lived experience of ADHD, and gained knowledge and insights from healthcare professionals, ensuring that tips and recommendations generated were feasible in the context of primary care in the United Kingdom. Additionally, our lived experience sample was relatively diverse, varying by gender, ethnicity, and geographical region. All lived experience research partners (except one) were able to attend the workshop, meaning they could discuss and share experiences, and help tailor content to suit diverse audiences. Unfortunately, our healthcare professional sample was less diverse. All healthcare professionals and researchers were from South West or Midlands. Given the variability of ADHD services in the United Kingdom, it would have been beneficial to have a broader range of geographic representation. In future research, sampling from a broad range of geographic locations and job roles within primary care may help improve workforce diversity in the samples.

Another limitation is that while we used purposive methods to achieve a diverse sample, to include people with a good understanding of research engagement, we focussed on research partners already involved with ADHD research at Exeter. While this supported co‐production principles of building strong and trusting relationships [24], and enabled richer data to be collected, it may have limited the generalisability of our findings. This could contribute to generated resources being less useful for a research‐naive population. Strengths of our approach are that research partners were already familiar with research, and likely to have been more confident contributing. They were also already familiar with each other. This is especially important as previous research indicates that online environments can slow down formation of trusting relationships [47].

A strength of this project was the involvement of stakeholders from design to dissemination of research. The study design was presented at RAG meetings where members were able to offer feedback on the methods. In addition, all research partners had the opportunity to help write this paper and share findings at a Primary Care seminar at the University of Exeter. This publication has been strengthened by co‐authorship from a multi‐disciplinary team including clinical, research and lived expertise and experience in ADHD, nursing, public health, primary care, computer science, and commissioning.

## 5 Conclusions

In this study, we highlight how co‐production methods can add valuable context to study findings and help researchers share learning in ways which maximise impact for a variety of audiences. Our co‐production workshops revealed that healthcare services are not only failing individuals with ADHD, but also general practice staff who are responsible for delivering care and often lack support when dealing with this long‐term health condition. Our recommendations focus on short‐term solutions to difficulties accessing care, but national policy updates are needed to address deeper challenges faced by this underserved group. We welcome the announcement by NHS England of an ADHD Taskforce and are hopeful that this research will contribute to and inform systems level change.

## Author Contributions

**Rebecca Gudka:** methodology, investigation, formal analysis, project administration, writing–review and editing, writing–original draft. **Anita Salimi:** methodology, investigation, formal analysis, writing–original draft, writing–review and editing. **Rachel Gaywood:** investigation, writing–original draft, writing–review and editing. **Dale Hendrick:** investigation, writing–review and editing, writing–original draft. **Kieran Becker:** project administration, investigation, writing–review and editing. **Oliver Medzinskii:** project administration, investigation, writing–review and editing. **Faraz Mughal:** supervision, writing–review and editing. **Jane Smith:** supervision, writing–review and editing. **Tamsin Newlove‐Delgado:** supervision, writing–review and editing. **Anna Price:** conceptualisation, funding acquisition, methodology, supervision, writing–review and editing.

## Ethics Statement

The project was given ethical approval by the Yorkshire and the Humber—Bradford Leeds Research Ethics Committee (Reference: 22/YH/0132). Informed consent was gained from all participants in line with Health Research Authority ethics.

## Conflicts of Interest

The authors declare no conflicts of interest.

## Supporting information

Supporting information.

Supporting information.

## Data Availability

The data that support the findings of this study are available on request from the corresponding author. The data are not publicly available due to privacy or ethical restrictions.
