# Supplementary material for: Co‐Producing Resources to Help Improve Access to Primary Care for Young People With Attention Deficit Hyperactivity Disorder
Source: Health Expect. 2025 Apr 29;28(3):e70200. doi: 10.1111/hex.70200 (PMC12041125; doi:10.1111/hex.70200)
Supplement: Supplementary file 1 — Supporting information. [file HEX-28-e70200-s001.pdf]

Appendix 1: Infographic and Workbook for participants

Mapping
ADHD services in
Primary Care

Exploring access to healthcare for neurodiverse young people

Work package 3: Co-production

Co-production workbook

Name:

Role(s):

Preferred pseudonym*:

*All participants will be pseudonymised in published reports/outputs from this work package. If you have a preferred pseudonym, please add it here and we will attempt to use this if possible. *Please ensure that this does not reveal your identity (i.e., do not use middle names or nicknames).*

Please review this workbook before the workshop and fill in as much information as possible. You can add to your thoughts during the workshop. At the end of the workshop please return your completed workbook.

Contents

Introduction i
- Aims and outputs i
- What is co-production? i
- How to use this workbook i

Establishing co-production ground rules + roles 1
- Beneficiaries 1
- Expertise 1
- Ground rules 1

Content/output priorities 2
- Content priorities 2
- Outputs 2

Section 1: Shared care prescribing 3
- Knowledge about Shared Care Agreements 3
- Shared care template (HP only) 3

Section 2: Reasonable adjustments in Primary care 4
- Template letter to general practice 4
- Suggested reasonable adjustments 4

Section 3: Practical support for ADHD 5
- Appointment checklists 5
- Non-pharmacological support 5
- Other tools 6

Section 4: What does ADHD look like? 7

Section 5: Information for health professionals 8
- Key messages for General Practitioners 8
- Policy makers 8
- Non-clinical staff 9

Looking into the future 10
- Things we may have missed 10
- Unanswered research questions 10
- Digital delivery 10

Thank you! 11

Introduction

Thank you for joining Work Package 3 of the Managing ADHD in Primary care (MAP) study. We have completed our national survey and interviews with ADHD stakeholders (e.g., people with lived experience, health professionals) from across England. In this work package, we're putting those findings and your expert opinion to use!

To read more about what we have done up to this point, click here.

Aims and outputs

Our aim is to **co-produce** evidence-based resources to improve the delivery of primary care for young people with ADHD, based on findings from the MAP studies. We aim to produce:

- **Resources with information for young people with ADHD and supporters**
- **Key messages and resources for primary care professionals (e.g., GPs) and commissioners**

What is co-production?

Co-production is a way of working that involves all key stakeholders: people using health services, supporters, practitioners, and researchers.

Co-producing a research project is an approach in which researchers, practitioners and the public work together, sharing power and responsibility from the start to the end of the project, including the generation of knowledge. – NIHR

Some of the key principles of co-production, laid out by the NIHR are as follows:

- **sharing of power** – the research is jointly owned and people work together
- **including all perspectives and skills** – make sure the research team includes all those who can make a contribution, encompassing the different types of expert
- **respecting and valuing the knowledge of all** – everyone is of equal importance
- **reciprocity** – everybody benefits from working together
- **building and maintaining relationships** – an emphasis on relationships is key

How to use this workbook

This workbook aims to provide structure to the co-production workshops. The prompts in the workbook have been identified from our research so far. As you progress, there are opportunities to reflect on questions, fill sections in, and add new content/ideas.

By the end of the co-production work, we will use the completed workbooks to produce resources that can be shared amongst young people with ADHD, their supporters, and healthcare professionals.

If you have any questions while you're completing the workbook, please feel free to reach out to the research team.

Establishing co-production ground rules + roles

This section aims to produce working guidelines for the workshop so that everyone feels they can contribute meaningfully. Reflect on the prompts and write down your thoughts.

Beneficiaries

What can I gain from this project? How can other people like me gain from this project? Who else stands to gain from this project?

[Enter your thoughts here]

Expertise

What am I an expert in? How will this expertise add to this project? Why are the different roles in this project important?

**Researcher**	**Health professional**	**Young person with ADHD/their supporters**
e.g., Researchers have knowledge in…	*e.g., HPs offer important insights into…*	*e.g., Young people provide useful thoughts on…*

Ground rules

What do I need from others to be able to take part in this project? How can I help others contribute?

[Enter your thoughts here]

Content/output priorities

The content of this workbook is based on priorities and outputs from MAP study findings. Let us know your thoughts on this by completing the activities below. You may want to revisit this page once you have completed the workbook.

Content priorities

Drag and drop to prioritise the section headers by importance. If you think we have missed anything, fill in the blank spaces and prioritise those too. Also feel free to add notes to each section, if you have preferred language or phrases to use.

Shared care prescribing

Information for health professionals

Reasonable adjustments in primary care

Practical support for ADHD

Digital Delivery

What does ADHD look like?

[Other]

**1 – Most important**	*[Drag and drop]*	*[Notes on this priority/section]*
**2**		
**3**		
**4**		
**5**		
**6**		
**7 – Least important**		

Outputs

The outputs we aim to achieve from this workbook are:

- **A downloadable resource for young people with ADHD and their supporters**
- **Posters/infographics about important features of ADHD**
- **Information for health professionals (including policy makers)**

Are there any other ways that we could use findings from the study findings to improve primary care provision?

[Enter your thoughts here]

Section 1: Shared care prescribing

In the MAP study, we found deficits and variation in shared care prescribing across the country. The below exercises will inform resources to help standardise shared care prescribing across England and provide useful resources for patients on facilitating the setting up of shared care agreements.

Knowledge about Shared Care Agreements

Patient knowledge

How would you describe shared care to someone with ADHD if they hadn't heard of it before?

[Enter your thoughts here] [The text box will expand when you start typing]

What do people with ADHD/their supporters need to know about shared care to navigate the processes with ease? Do you have any top tips on troubleshooting shared care *(e.g., what are my rights for prescribing from my GP?)*

[Enter your thoughts here]

Practitioner knowledge

> *GPs are not legally obliged to take on shared care. There are also lower rates of prescribing for patients with private diagnosis (49%) than NHS diagnosis (90%).*

What do health practitioners need to be more confident prescribing medication under shared care agreements?

[Enter your thoughts here]

Shared care template (HP only)

Do you have a shared care template that you currently use for adult ADHD prescribing? Are there any changes you would make to this template to make it easier to use etc?

<<Insert shared care agreement template here>>

Section 2: Reasonable adjustments in Primary care

Our qualitative study found that patients would often benefit from reasonable adjustments to accessing care, such as being able to email the surgery to request appointments.

Template letter to general practice

To help patients make requests for reasonable adjustments, we would like to create a template letter that patients can fill in with their specific needs/adjustments. Help us by providing some text that would be useful to include in the template letter.

Patient name
Patient address
Patient phone number

Name of GP
Address of surgery

Date

Dear [practice name] administration,

[Enter your thoughts here]

Suggested reasonable adjustments

Add to the list below of reasonable adjustments that people with ADHD might find useful. This list can then be used as a useful guide/checklist for patients to add to the letter they send to their GP.

Reasonable adjustment	Why would this be useful?
e.g., Adding preferences/additional information to their Summary Care Record	*e.g., It means patients would not have to explain certain preferences to every clinician they see*
e.g., Allowing patients to have relatives/supporters to act as a proxy contact	
[Enter more reasonable adjustments here]	

Do you have a template letter from your practice that can be used to request a legal proxy? Are there any changes that could be made to make this form simpler for patients with ADHD?

<<Insert proxy letter template here>>

Section 3: Practical support for ADHD

We would like to create practical tools for patients to use when accessing primary care. Below we have suggested some tools which we think might be useful. Please add to them or come up with your own ideas for useful tools.

Appointment checklists

Our findings show that patients struggle to remember what to ask/say/do in an appointment with their GP. Taking checklists into their appointments might help alleviate these difficulties. Write below what you think would be useful to go on these checklists.

Appointment about initial concerns checklist

What do I need to ask/say/do?	Notes	Done?
e.g., Tell my Dr about my symptoms.	*e.g., My symptoms are...*	☐
[Enter your thoughts here]		☐
		☐
		☐

Medication/monitoring appointment checklist

What do I need to ask/say/do?	Notes	Done?
[Enter your thoughts here]		☐
		☐
e.g., Ask to arrange my next monitoring appointment.	*e.g., 6 months from today is __/__/____.*	☐

Are there other appointment types that would be useful to have checklists for?

Non-pharmacological support

Since waiting times for adult ADHD services are 2+ years long in many regions across England, accessing mental health and wellbeing support via primary care is crucial for people who are on the wait lists for diagnosis/treatment of ADHD.

What would be useful to be able to provide to your patients who are on the waitlist?

e.g., Referral to social prescribing support or mental health wellbeing worker; signposting to specific resources.

[Enter your thoughts here]

What would be useful to be able to provide to your patients once they're receiving treatment for ADHD?

[Enter your thoughts here]

Other tools

Do you have any other ideas for practical tools that might help young people with ADHD when they are accessing primary care? Feel free to get creative with how these tools might look and make suggestions on how to present these to patients.

[Enter your thoughts here]

Section 4: What does ADHD look like?

We would like to produce some posters/infographics on different presentations of ADHD. These may be used to help people recognise their own symptoms, or for health professionals to recognise "unusual" presentations of ADHD. Using the box below, get creative and think about what these posters could look like (or feel free to draw on paper and send in photos to us if that's easier).

Section 5: Information for health professionals

Key messages for General Practitioners

From the research findings, we want to produce a short and easy to read information for GPs to try and interest more in neurodiversity and treating people with ADHD.

What are the 4 most important key messages from this research to tell GPs?

-
-
-
-

Policy makers

From MAP study findings, barriers to accessing care that were repeatedly brought to researchers' attention were complex processes/systems to book appointments and re-order repeat prescriptions and lack of access to non-pharmacological ADHD support.

Please help us draft a letter to commissioners and Primary Care Networks (PCNs) to emphasise the importance of this issue and suggest ways these systems might be improved. Use your own experiences to inform this message, and any language preferences you have.

Please add to/change the letter below using your own experience and expert opinion.

Dear Commissioners and PCNs,

Recent findings from the Managing ADHD in Primary care (MAP) study have highlighted massive gaps in the healthcare system for young people with ADHD, aged 16-25. The study has ben conducted between 2020-2024, comprising of a national survey and qualitative study of young people with ADHD, their supporters and health practitioners.

This research shows that:

Insert key important findings to share here.

These findings mean that there is not equitable access to primary care for young people with ADHD in England. To meet the legal requirements of the Equality Act (2010) we recommend some feasible changes to primary care in England.

Please enter your proposed changes to primary care structures/systems here:

- *E.g., Direct access to social prescribers/occupational therapists instead of requiring referrals via GPs*
- *E.g., Allowing patients to access an online appointment booking system*
-

These changes would enable young people with ADHD to:

How would these changes improve access?

In addition, they would be beneficial to the wider population and GP surgeries in general because:

Would these changes have benefits for people other than those with ADHD? Would thye improve access generally?

Would these changes help surgeries in a wider sense? (e.g., they may make processes quicker for admin teams and doctors, thus releasing resources?)

Non-clinical staff

We would like to produce some resources for non-clinical staff (e.g., receptionists and administrators) in GP surgeries to raise awareness about ADHD and how this might affect a patient's ability to book and attend appointments.

Is there anything that you would like non-clinical staff in general practices to know?

[Enter your thoughts here]

Looking into the future

Things we may have missed

If there are things that you think should be included in the outputs from this research, but you could not see a place to add them in the above sections, please let us know here.

[Enter your thoughts here]

Unanswered research questions

Is there anything from the MAP study that you feel has been left unanswered? Has it brought up any new research questions? Is there anything you think still needs investigating?

[Enter your thoughts here]

Digital delivery

We are currently working on plans for a future research project to deliver a Digital Health Intervention for young people with ADHD on the waiting list to see specialist care. We have started to plan the content that we would like to deliver, and how we might deliver it (e.g., video clips, gamification). If you have any thoughts on the framework below, please leave comments or add rows and let us know what you think.

<table>
<tr><th>Content</th><th>Mode of delivery</th><th>Notes/Thoughts</th></tr>
<tr><td>Self-management tools</td><td rowspan="4">Video/Audio clips</td><td></td></tr>
<tr><td>Psychoeducation</td><td></td></tr>
<tr><td>Peer stories on becoming an adult with ADHD</td><td></td></tr>
<tr><td>Condition specific health information</td><td></td></tr>
<tr><td>Self-management tools</td><td rowspan="10">Text (with images/cartoons)</td><td></td></tr>
<tr><td>Psychoeducation</td><td></td></tr>
<tr><td>Social Prescribing, linking with community resources</td><td></td></tr>
<tr><td>Understanding pathways to care, and different healthcare professional roles and responsibilities</td><td></td></tr>
<tr><td>Accessible summary of NICE guidance</td><td></td></tr>
<tr><td>Disability rights, incorporating healthcare, education, and workplace support</td><td></td></tr>
<tr><td>Condition specific health information (including managing co-morbidities)</td><td></td></tr>
<tr><td>Signposting to external resources (peer networks, websites)</td><td></td></tr>
<tr><td>Site navigation page</td><td></td></tr>
<tr><td>Understanding pathways to care, and different healthcare professional roles and responsibilities</td><td rowspan="3">Gamification (quizzes, points for content covered)</td><td></td></tr>
<tr><td>Accessible summary of NICE guidance</td><td></td></tr>
<tr><td>Disability rights, incorporating healthcare, education, and workplace support</td><td></td></tr>
</table>

Condition specific health information (including managing co-morbidities)		
Self-management tools		
Self-management tools	Graphs displaying personalised data over time (including diary)	
Symptom Tracking (including Health and wellbeing)		
Self-report of self-care		
Medication information and management tools	Email messages & reminders	
Symptom tracking		
Self-report of self-care		
Peer support	Integrated internal peer network	
Self-esteem scale	Questionnaires (optional – for research data)	
ADHD symptom scale		
Wellbeing scale		
PhQ9		
Quality of life scale		

Thank you!

Thank you for completing this workbook! You may now want to go back to the prioritisation task from the start. When you are finished, please send the workbook back to us at the MAP team so that we can get to work making resources!

As part of the co-production work, we would like to offer you all the opportunity to be involved in how we share these findings. This may involve things like helping us to write papers and deliver presentations.

Please indicate with an *x* whether you would like to be involved in any of the below activities.

**Activity**	**I would like to take part**
Contributing as an author to a research paper in a scientific journal	
Presenting the findings/outputs at a seminar at the University of Exeter	
Presenting findings/outputs at an external conference	
Sharing findings with my own networks	

If you have any feedback about these workshops, please reach out to a member of the research team.

KEY MAP STUDY FINDINGS

University of Exeter

Mapping ADHD services in Primary Care

As you may already know, the MAP study has previously conducted a national survey and an interview study about ADHD provisions in England.

This infographic summarises some of the key findings from these studies

PATIENTS JOURNEY

45% commissioners confirmed **2+ year waiting times** for adult mental health services for ADHD (40% across stakeholder groups)

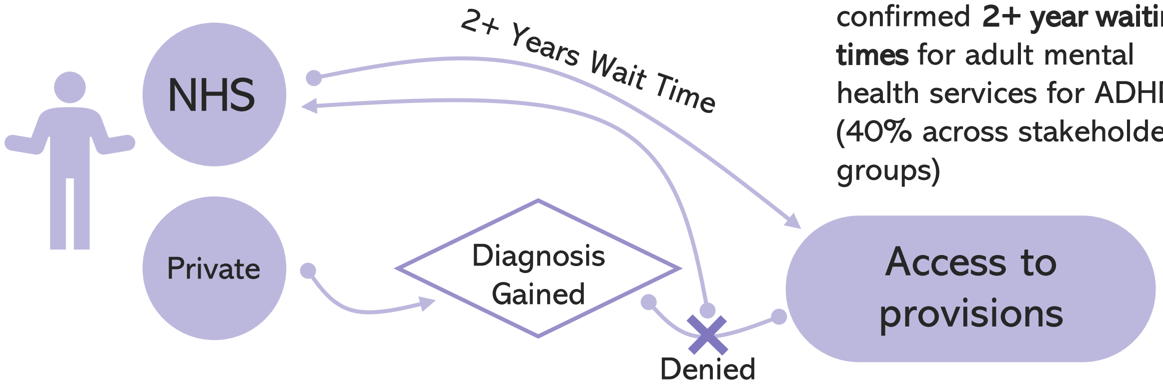

Additionally, health professionals reported **higher rates of prescribing** for patients with an **NHS diagnosis** (90%) than patients with a **private diagnosis** (45%).

BARRIERS TO ACCESSING CARE

Participants reported multiple challenges in accessing support for their ADHD, including:

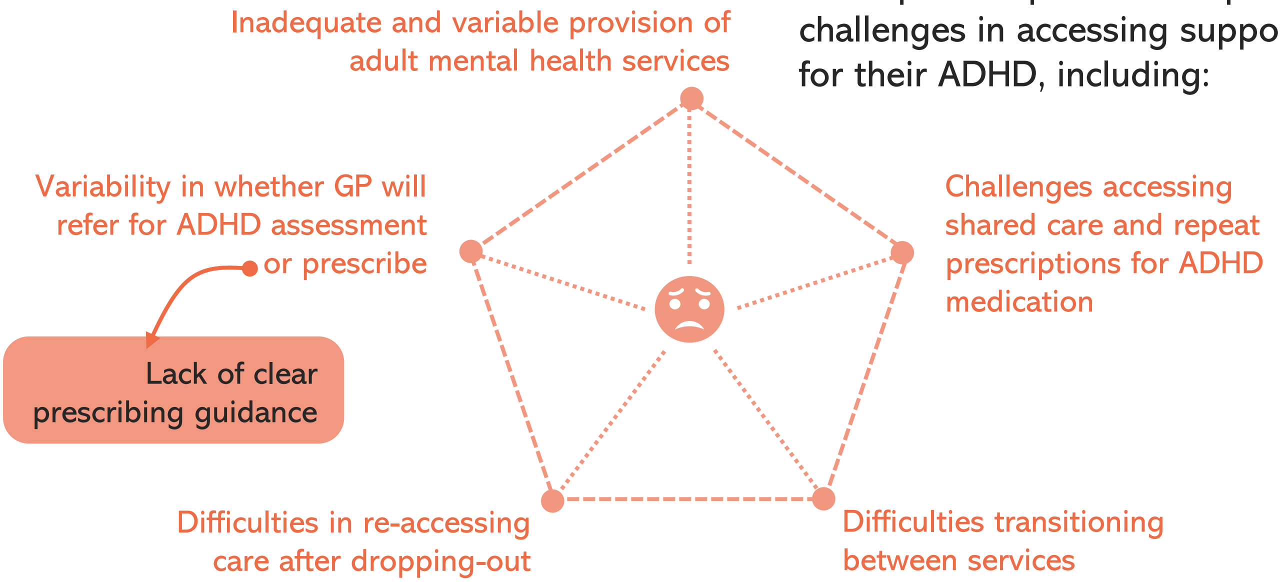

79%
health practitioners reported **available adult mental health services** (despite 100% commissioners reporting AMHS availability)

We found variable reported rates of prescribing per region
London (83%)
Midlands (56%)
South-West (55%)

Only
35.3%
commissioners reported that there was a mental health practitioner available to patients with ADHD in their local area.

These findings from the quantitative study demonstrate the need to support **equitable healthcare** for young people with ADHD and provide resources to streamline processes in Primary Care

"Here services are limited or unavailable, it is often the patients without family support or resources to enable private healthcare that are most negatively impacted."
Dr Anna Price, 2024

Our qualitative findings support the national survey results. Below are some examples of our key findings.

People with lived experience were often frustrated with how **complicated it was to use services** due to complex appointment booking processes. In addition, **medication prescribing and monitoring** was patchy and sometimes resulted in gaps to receiving medication.

"I have basically no communication with [my doctors] while I have the repeat prescription… Once in a blue moon, doesn't seem to have a pattern, they'll not fill it, and [say], "Oh, you actually need to come in, or you need to do your blood pressure and weight."
Young Person

Stakeholders reported **variation in ADHD care,** between regions, practices and individual practitioners. One GP reported that often, university practices have good mental health provision for patients with ADHD and may serve as a good example for other practices to follow for **reasonable adjustments in practices**.

"Within the university setting there's often a very strong welfare provision as well as a counselling service if needed, so students here are often very well supported."
GP

Many participants acknowledged that primary care are under pressure to take on more responsibility from secondary care and **resources are stretched thin**.

"It becomes something else that once upon a time was managed by secondary care, and it suddenly becomes a primary care thing. That's great in many ways, but it's not great in primary care because we're picking up something else from secondary care."
Practice Manager

DIGITAL RESOURCE DEVELOPMENT

Participants report that digital resources are currently limited, and not always specific to the needs of people with ADHD.

Information must be reliable and verified

Information must be accessible (multi-modal, multi-lingual)

Information and content must be *neurodiverse led*
