# Supplementary material for: Co‐Producing Resources to Help Improve Access to Primary Care for Young People With Attention Deficit Hyperactivity Disorder
Source: Health Expect. 2025 Apr 29;28(3):e70200. doi: 10.1111/hex.70200 (PMC12041125; doi:10.1111/hex.70200)
Supplement: Supplementary file 2 — Supporting information. [file HEX-28-e70200-s002.pdf]

Mapping ADHD services in Primary Care

ADHD for patients

The NHS is under pressure. Doctors have more and more responsibilities, but we understand that not being able to access care can be frustrating.

This leaflet contains some top tips from people with ADHD, healthcare professionals and researchers on how to access care if you're finding it hard.

NHS England has recognised the crisis in ADHD healthcare provision and has announced an ADHD Taskforce to look at attention deficit hyperactivity disorder (ADHD) service provision and its impact on patient experience.

Glossary of terms

Mapping ADHD services in Primary Care

In this document, we refer to different healthcare professionals who may be involved in your care.

- **GP** – general practitioner
- **Specialist** – usually a psychiatrist, this doctor will have expert training in ADHD and will be responsible for your assessment and getting medication set up
- **Healthcare provider** – a group or organisation who provides care. **Private providers** take payment for services (e.g., the NHS is a free healthcare provider, Psychiatry UK is a private provider)

We also refer to different stages in the ADHD care pathway.

- **Referral** – when your GP sends you to a specialist for healthcare (e.g., your GP will refer you to a specialist for your diagnostic assessment)
- **Assessment** – An appointment with a specialist to determine whether or not you have ADHD.

  The assessment may include:

  - a physical examination, which can help rule out other possible causes for the symptoms
  - a series of interviews with you or your child
  - interviews or reports from other significant people, such as partners, parents and teachers

  See the NHS website for more information about what to expect.
- **Diagnosis** – after your assessment, if your provider thinks you have ADHD, they will give you a diagnosis and they will help you manage/treat your ADHD. If you do not have ADHD, you won't receive a diagnosis, but your provider may still help you manage any symptoms you are having and help you decide what to do next.

ADHD roadmap

Mapping ADHD services in Primary Care

This diagram shows the stages that you might go through when you seek an ADHD diagnosis and treatment, and some of the barriers people commonly experience. Throughout this leaflet, you will find top tips for overcoming these barriers from our expert participants.

We have split the roadmap into three stages, each with a colour which corresponds to pages about that stage throughout the document.

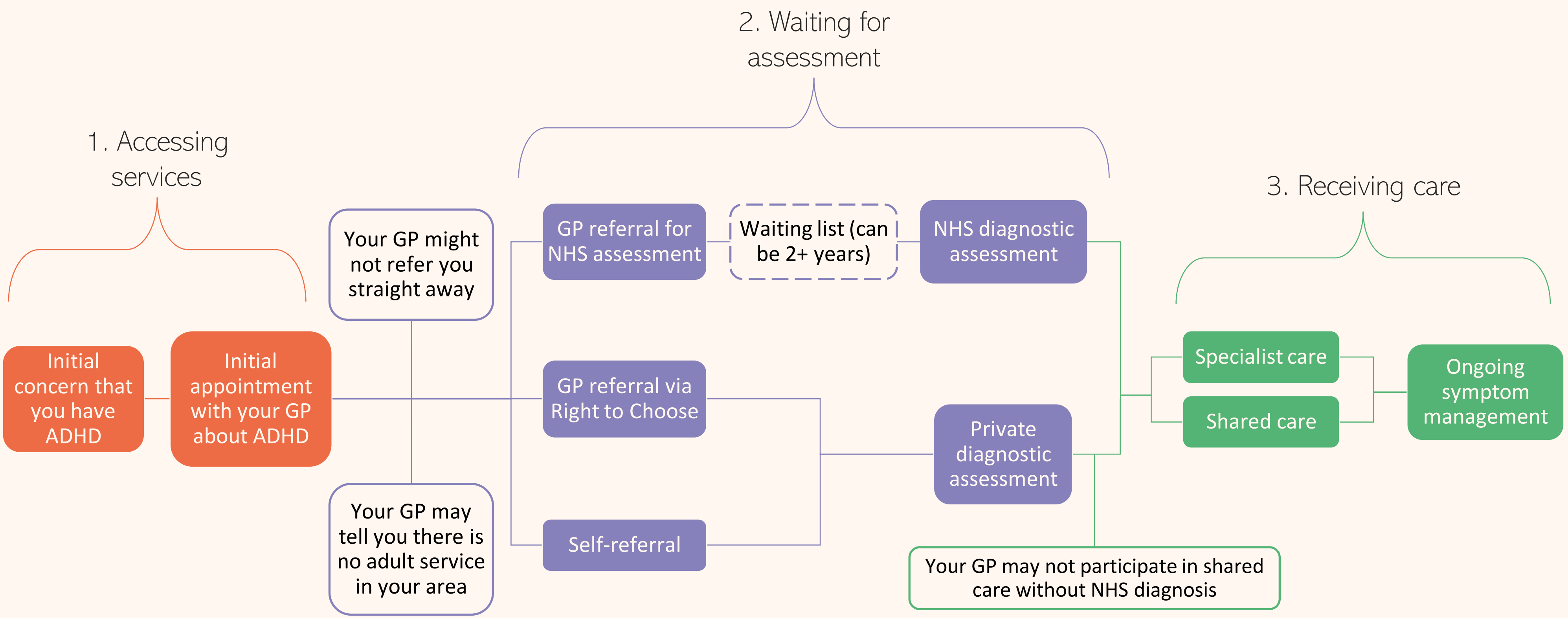

1. Accessing services

Mapping ADHD services in Primary Care

Reasonable adjustments

It is a legal requirement in the UK to make healthcare accessible to all

Some people with ADHD require help to make attending GP appointments easier

This could be things like:

- Text reminders for appointments where possible
- Longer appointments if someone needs more time with a doctor/nurse

"A lot of ADHD people have anxiety, so for people to be aware in a medical setting. They're quite likely to be anxious because they tend to overthink things … I think that should be flagged to doctors."

- Participant with lived experience of ADHD

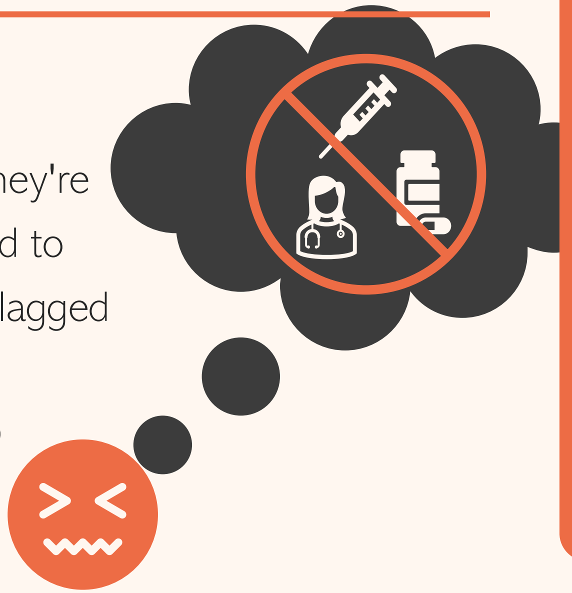

What would make it easier for you to access and attend GP appointments?

1. Use the checklist on the next page to identify reasonable adjustments that might help you
2. Make an appointment with your GP to discuss them
3. During your appointment, you can ask your GP to create an alert in your file so that GP staff (receptionists, doctors, nurses) can see which adjustments to put in place

Reasonable adjustments - Checklist

Mapping ADHD services in Primary Care

What would make it easier for you to access and attend GP appointments?

1. Use the checklist to identify reasonable adjustments that might help you
2. Make an appointment with your GP to discuss them
3. During your appointment, ask your GP to create an alert in your file so that GP staff can see which adjustments to put in place

Additional support		Accessible information	
I require appointment reminders		I prefer written communication	
I would like my carer at appointments		I prefer verbal communication	
I have anxiety related to clinical settings		Please contact me via my carer	
I require minimal waiting time between arrival and being seen		Please contact me via [email, letter, short message service/text message, telephone]	
I require an extended appointment			
I have a preference for a female/male doctor		I require information in Easy Read format	
I require the first / last appointment in the day		Please be aware that I have difficulty processing information	
I require a priority appointment		**Adjustment to the environment**	
**Bespoke adjustments**		I am sensitive to bright lights/loud noise	
e.g., I may find it difficult to be on time for my appointments		I require a low light and low noise room to wait for my appointment in	

2. Waiting for assessment

Options for ADHD care

Mapping ADHD services in Primary Care

NICE guidelines for ADHD service provision state that *all patients have a right to an ADHD assessment.*

There are different options for accessing ADHD care in the UK

1. **NHS assessment**
    - If your GP says there is **no ADHD Assessment service in your area,** you can ask them to help you access a service elsewhere using an Individual Funding Request (find out more about this here.)
    - If your GP advises you of long wait lists, you may consider **options 2** or **3**

2. **Right to Choose**
    - In the UK, everyone has a legal right to choose your mental healthcare provider
    - You can choose a provider that has a contract with the NHS and ask your GP to refer you to them – this is called the "Right to Choose" process

3. **Private assessment**
    - You can see a private provider to receive your ADHD care, however this can be expensive.

See the next page for pros and cons of each of the above options. If you are struggling to decide between options, speak to your GP, people who you trust around you, or contact a charity such as ADHD UK who may be able to help.

Options for ADHD care

NHS care		Right to Choose		Private care	
Pros	Cons	Pros	Cons	Pros	Cons
Free – you do not have to pay for services	Long wait times to receive diagnostic assessment	Free – you do not have to pay for services	Can still have long wait times for diagnostic assessment	Shorter wait times for diagnostic assessment	You have to pay for services which are can be expensive (see here for an estimate of fees)
GP will sign Shared Care Agreement	Access to medication **post-diagnosis** can be slow	GP likely to sign Shared Care Agreement	Contracts with NHS can change, so Shared Care Agreements may be transferred to new provider	Faster access to medication post-diagnosis	Some GPs are not supported to take over care and can not sign a Shared Care Agreement

If your GP won't sign your Shared Care Agreement because it's with a private provider, ask them if they will refer you get an NHS assessment and accept the Shared Care Agreement while you wait.

3. Receiving care

Mapping ADHD services in Primary Care

Shared care – What is it?

Shared Care Agreement (SCA) - An agreement between the specialist who diagnoses you and trials your medication, and the GP who will prescribe your medication going forward. The SCA specifies the type and dose of medication you take.

- Your specialist and GP both sign the SCA to agree to *share responsibility of your ADHD care*

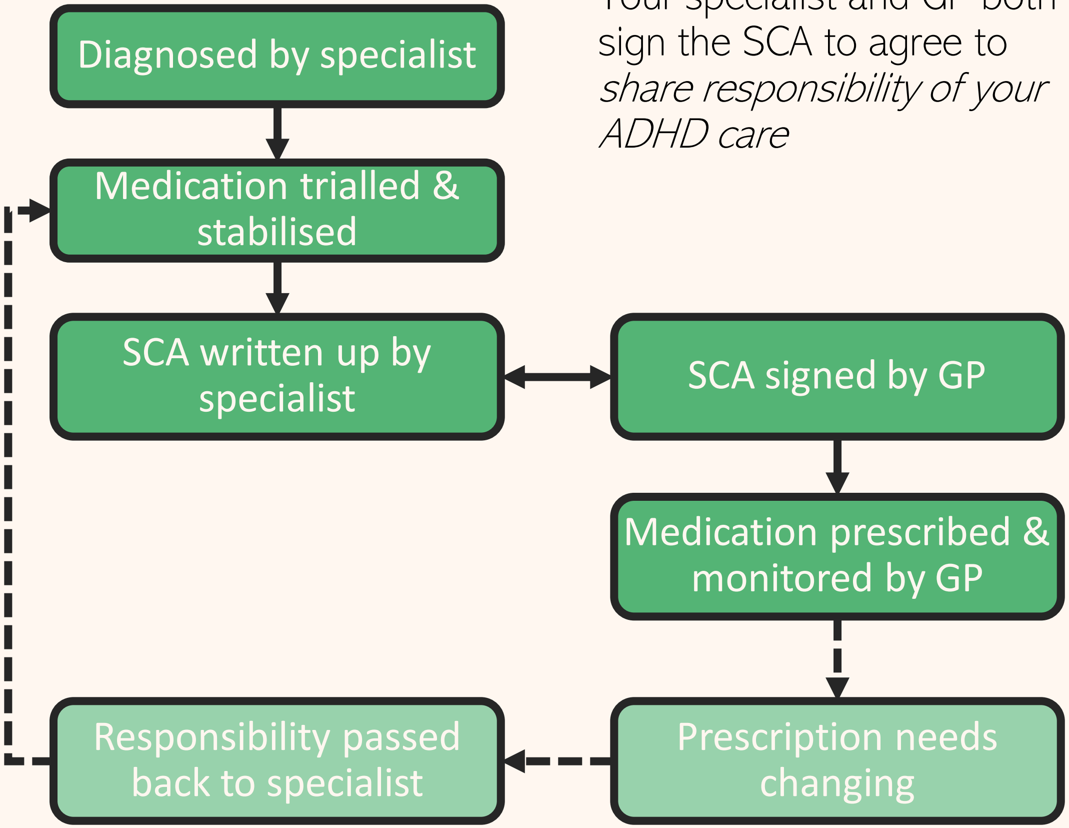

Your GP does not legally have to agree to shared care. There are a few reasons why a GP may not agree:

- They may not have the clinical capacity to sign or may feel unsupported to share responsibility for your care
- If you have a private specialist who has initiated the SCA:
  - They may have recommended medication that the NHS/your GP is not allowed to prescribe
  - Your GP may not have a prior contract with your private provider and therefore may not be allowed to enter an SCA with them

Shared care – Top tips

Mapping ADHD services in Primary Care

What to do if your SCA is too narrow, making accessing medication hard?

E.g., the specialist recommended 20mg tablets of Concerta in the SCA. However, this meant that if there was a shortage of 20mg tablets, or Concerta was out of stock, the GP cannot prescribe 2x10mg or other brands of medication.

You can ask your specialist to make the SCA as broad as possible. This could include notes such as "if no 20mg available, use 2x10 mg." or "if [medication brand] is unavailable use [alternative brand]".

What to do if you're thinking about getting a private assessment?

Try speaking to your GP or the practice manager before you seek a private assessment to ensure your GP will prescribe under an SCA with your chosen provider.

- "If I get a diagnosis from [provider], will you be willing/able to enter an SCA with them?"
  - If your GP says they will not sign an SCA with the private provider, you could try to find another GP in your area, or you can add this to a pros/cons list when deciding whether to use a private specialist

What to do if your GP won't sign your SCA?

NICE guidance states that your GP should "make appropriate arrangements for continuing your care" even if they do not agree to your SCA

- Meeting with your GP to ask about their decision and explain the impact this will have on your life
- Ask if they would consider putting you on the NHS waitlist and provide shared care while you are waiting
- You can see more about these options here: https://www.adhdadult.uk/shared-care/
